# Dissolution Behavior of Eutectic Carbides in Medium-Alloy Steels for Heavy Forgings

**DOI:** 10.3390/ma16206763

**Published:** 2023-10-19

**Authors:** Yu Ji, Tingting Xu, Caiyan Zhao, Guangyao Chen, Hongshan Zhao, Chundong Hu, Han Dong

**Affiliations:** 1 School of Materials Science and Engineering, Shanghai University, Shanghai 200444, China; jiyu@shu.edu.cn (Y.J.); huchundong99@163.com (C.H.); donghan@shu.edu.cn (H.D.); 2 Zhongyuan Special Steel Co., Ltd., Jiyuan 459000, China; xuting_1@163.com (T.X.); zcy8775@163.com (C.Z.); 3 Zhejiang Institute of Advanced Materials, Shanghai University, Jiaxing 314100, China

**Keywords:** heavy forging, high-temperature diffusion, Cr-Ni-Mo-V alloy steel, eutectic carbides, hardness

## Abstract

In this study, we investigate the dissolution behavior of eutectic carbides in heavy forgings. High-temperature diffusion treatment was conducted on 35Cr3Ni3MoVW2 (MoVW2) and 35Cr2Ni3MoV (MoV) steels at 1230 °C for a duration ranging from 0 to 100 h. The dissolution of eutectic carbides and its effects on the microstructure and hardness of the steels were characterized and analyzed via SEM+EBSD, ImageJ, and Thermo-Calc. The results show that the coarse eutectic carbides in both steels gradually dissolved. The distribution and morphology tend to be uniform and spherical, respectively. For holding 50 h, the hardness of both steels significantly exhibited an increasing trend, and it was attributed to the combined effects of solid solution strengthening. Thermodynamic calculations indicated that the higher W content in MoVW2 steel promoted the precipitation of M_6_C eutectic carbides. Moreover, both MoVW2 and MoV steels exhibited the precipitation of M_7_C_3_ eutectic carbides in the final stage of solidification, facilitated by the enrichment of C and Cr in the liquid steels.

## 1. Introduction

Heavy forgings are widely used in fields such as nuclear power rotors and high-pressure vessel equipment. They are the foundational parts of significant technological equipment, and the quality is an important indicator for evaluating China’s manufacturing level [1]. To date, the heavy forgings used in high-end equipment have been made from Cr-Ni-Mo-V alloy steels. However, due to the large size of these forgings, the uneven temperature distribution can cause the formation of carbon-rich regions and areas enriched with alloy elements during the final solidification. Thus, it could further cause the formation of eutectic carbides [2]. Generally, the carbide, which acts as an important alloy phase, can directly influence the high-temperature strength and heat resistance of alloy steels through quantity, morphology, size, and distribution [3]. Coarse eutectic carbides not only disrupt the continuity of the steel matrix but also facilitate the formation of cracks, thereby reducing the workability and usability of alloy steel [4,5]. Therefore, it is important to investigate the characteristics of carbides in steel forgings as well as the controlling factors.

Recently, researchers conducted several studies on how to control the formation of eutectic carbides in alloy steels. A study by Zhang et al. revealed that M_7_C_3_ eutectic carbides could be refined by adjusting the sequence of Nb and Ti additions and optimizing the element distributions in (Nb,Ti)C [6]. Li et al. showed that it was possible to obtain eutectic carbides with smaller size and more uniformly dispersed distribution by increasing the solidification cooling rate [7]. Qu et al. revealed that the morphology of eutectic carbides could be refined by using rare-earth metals [8]. By comparison with techniques such as adjusting steel smelting processes and compositions, high-temperature diffusion treatment could be considered as an effective way to reduce the content of eutectic carbides and improve the thermal-processing performance of steels. This is primarily because this treatment did not alter the alloy composition or production procedures [9].

High-temperature diffusion treatment for alloy steels was primarily influenced by heating temperature and holding time. A study revealed that a suitable heat-treatment temperature for carbon steels and alloy steels was about 1100~1200 °C and 1200~1300 °C, respectively [10]. Also, increasing the heat-treatment temperature was more effective at improving structural uniformity and reducing eutectic carbides than extending the holding time [11]. Previous studies only focused on the effect of the heating temperature on the diffusion processes for eutectic carbides. There are very few investigations into the holding time [12,13]. Therefore, the effect of the holding time on eutectic carbides in alloy steels still requires in-depth theoretical and experimental research.

In this study, the effect of high-temperature diffusion time on the microstructure and hardness of as-forged Cr-Ni-Mo-V alloy steels was studied, and the precipitation behavior of the eutectic carbides was also investigated using Thermo-Calc v2015a software. The aim is to reduce the eutectic carbides in the steels and enhance their high-temperature performance. The findings provide a valuable reference and guidance for the industrial production and utilization of alloy steels.

## 2. Materials and Methods

The experimental Cr-Ni-Mo-V steels were obtained from industrial productions of heavy forgings. The chemical compositions of the steels were 35Cr3Ni3MoVW2 (referred to as MoVW2) and 35Cr2Ni3MoV (referred to as MoV), as shown in Table 1. After forging, the steels were subjected to high-temperature diffusion annealing at 1230 °C for 0, 10, 20, 30, 50, 70, 80, and 100 h, separately. Then, the steels were cooled in the air. The steel specimens were sectioned using a cutting machine (Biaole Abrasimet M, Buehler, Lake Bluff, IL, USA), polished using the abrasive papers, and subsequently etched in a solution of nitric acid and ethanol (with a nitric acid concentration of 4 wt.%) for a duration of 10 to 15 s. Simultaneously, 10 mm × 10 mm × 5 mm samples were taken, and vibratory polishing was performed using 0.02 µm colloidal silica suspension for microstructural analysis (EBSD) (Bruker QUANTAX EBSD 400i e-Flash^FS^, Bruker, Billerica, MA, USA). This analysis was further complemented with AZtecCrystal v2.1 software for post-processing orientation relationships within the microstructure.

The metallographic microstructure of the steel specimens was observed using an upright fully automated metallurgical microscope (Carl Zeiss Axio Imager.M2m, Carl Zeiss, Jena, Germany), while the microstructural morphology was examined using a field emission scanning electron microscope (FEI Apreo 2S HiVac, Thermo Fisher Scientific, Waltham, MA, USA). The ImageJ 1.53c software was employed for statistical analysis of the volume percentage and diameter dimensions of the eutectic carbides in the steels (with 20 observations of 5000× fields for each sample). The hardness of the steels was detected using a semi-automatic Brinell hardness tester (Wilson WHBH3000, Bruker, Billerica, MA, USA) with 750 kg load. Additionally, Thermo-Calc thermodynamic software using the Scheil model was utilized to investigate the precipitation behavior of eutectic carbides in the steels after the non-equilibrium solidification.

## 3. Results and Discussion

**Figure 1:**
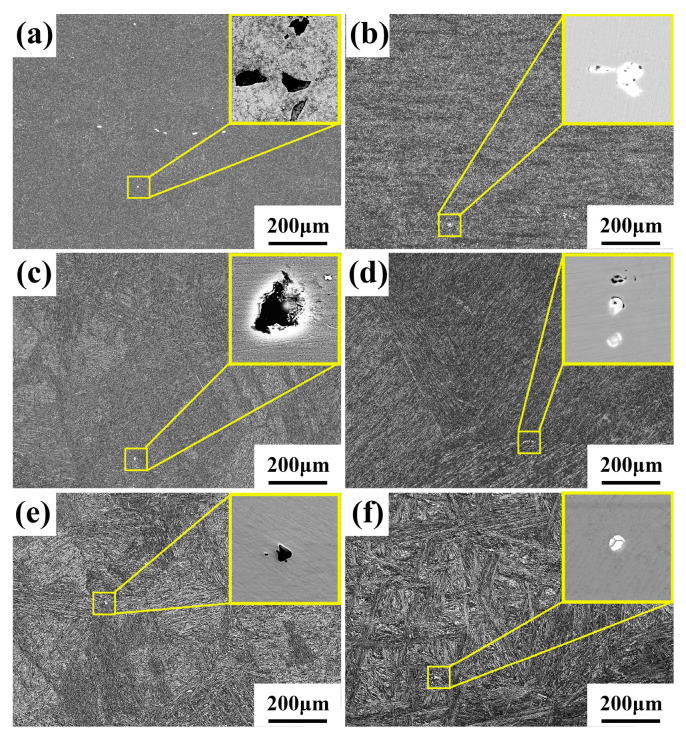
SEM images of eutectic carbides in MoVW2 and MoV steels after holding at 1230 °C for 0, 50, and 100 h. (**a**,**c**,**e**) MoVW2, 0, 50, 100 h; (**b**,**d**,**f**) MoV, 0, 50, 100 h.

Figure 1 shows the microscopic images of MoVW2 and MoV steels after holding at 1230 °C for 0, 50, and 100 h. From Figure 1a,b, it can be seen that the average diameter of the eutectic carbides in MoVW2 and MoV steels were 14.25 and 8.57 μm after holding for 0 h, and the eutectic carbides in the steels exhibited an uneven distribution and irregular shapes. As the holding time increased to 50 h, the morphology of eutectic carbides transformed into a chain-like or block-like structure, and its sizes exhibited a decrease trend, as shown in Figure 1c,d. It was for this reason that the heat treatment for the steels could cause the dissolution of eutectic carbides. Upon extending the holding time to 100 h, the average diameter of the eutectic carbides in MoVW2 and MoV steels was 5.02 and 5.44 μm, respectively. Additionally, the coarse chain-like or block-like eutectic carbides were mostly eliminated and transformed into a spherical shape, as shown in Figure 1e,f. A study by Mao showed that the spherical carbides had a minor impact on the properties of the steels [14]. It indicated that a long holding time for the steels contributed to the dissolution of the carbides and reduced the effects of large-sized and irregular eutectic carbides on the steels.

**Figure 2:**
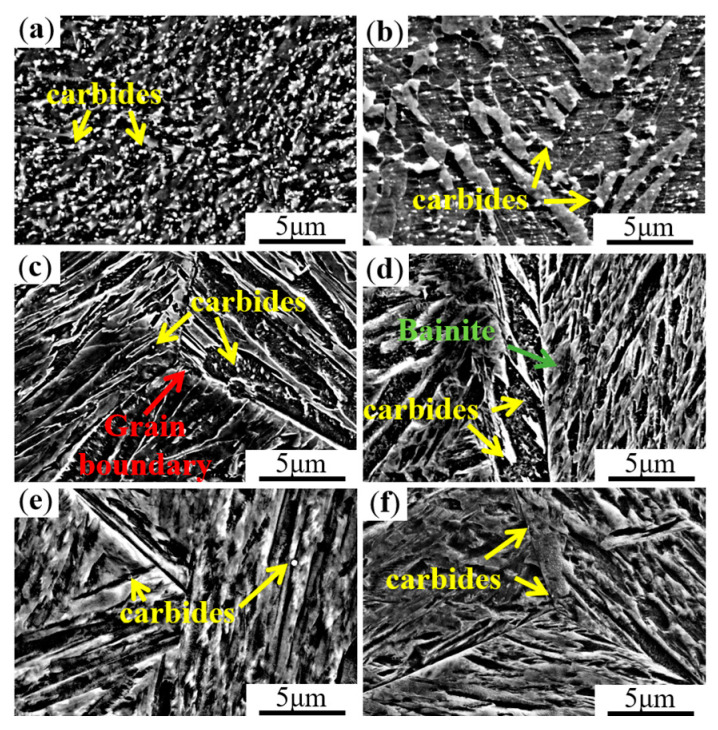
SEM images of MoVW2 and MoV steels after holding at 1230 °C for 0, 50, and 100 h. (**a**,**c**,**e**) MoVW2, 0, 50, 100 h; (**b**,**d**,**f**) MoV, 0, 50, 100 h.

Figure 2 shows the SEM images of MoVW2 and MoV steels after holding at 1230 °C for 0 h, 50 h, and 100 h. From Figure 2a,b, it can be seen that the carbides in MoVW2 steel exhibited a uniformly distributed granular pattern after holding for 0 h. However, in MoV steel, the carbides exhibited an irregular shape and uneven distribution. After holding for 50 h, the original austenite grains were coarse, and they could suppress the nucleation of the ferrite. Additionally, a small amount of bainite could also be observed during the cooling [15,16]. From Figure 2c,d, it can be seen that the size of the grains in the steels increased with the increasing holding time, and the carbides along the grain boundaries gradually disappeared. It was for this reason that the dissolution of carbides weakened the pinning effect on the grain boundaries, facilitating the growth of grains. As the holding time increased to 100 h, it was difficult to observe the carbides, indicating that they were mostly completely dissolved, as shown in Figure 2e,f.

**Figure 3:**
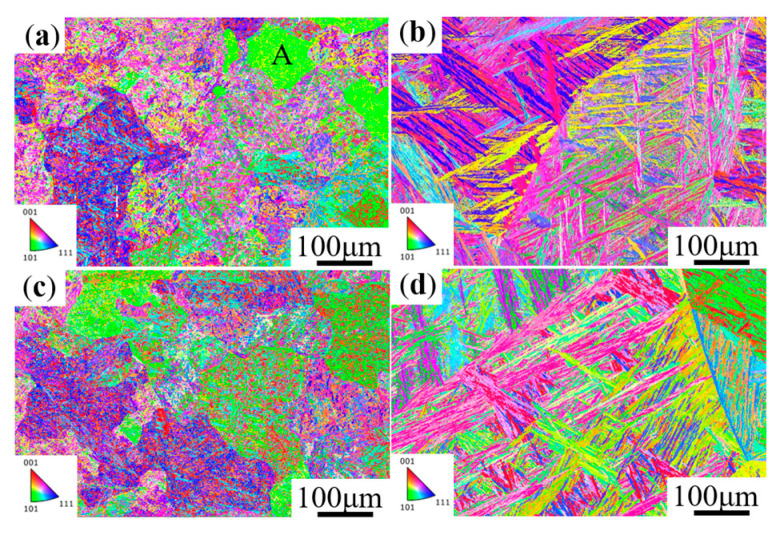
IPF images of MoVW2 and MoV steels after holding at 1230 °C for 0 and 100 h. (**a**,**b**) MoVW2, 0, 100 h; (**c**,**d**) MoV, 0, 100 h.

Figure 3 shows the inverse pole figure (IPF) of MoVW2 and MoV steels for holding at 1230 °C for 0 and 100 h. From Figure 3a, it can be seen that grain A had a (101) orientation, and other grains had a relatively single orientation. After holding for 100 h, multiple orientations appeared, because the small grains were absorbed by the large grains during the high-temperature diffusion process, resulting in the grain coarsening, as shown in Figure 3b [17]. Apparently, the growth rates of various grains were uneven, resulting in the appearance of mixed crystallization [18]. Additionally, a long holding time was effective in the generation of a fine needle-like and disordered microstructure in the steels.

**Figure 4:**
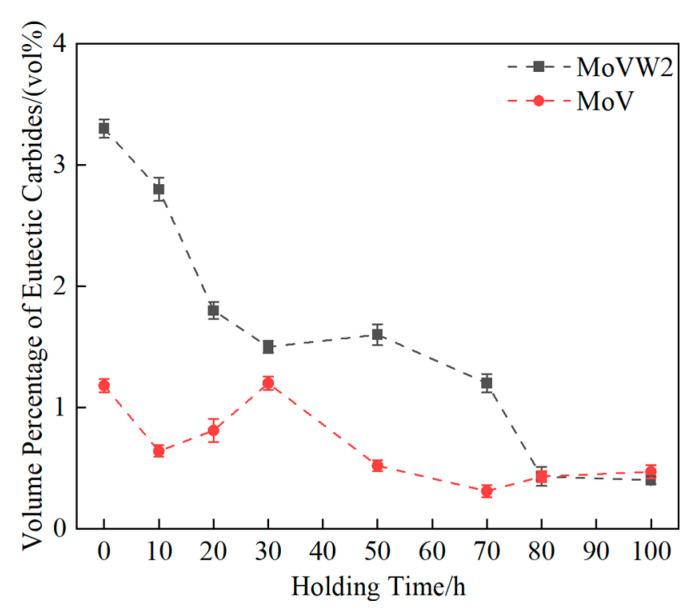
Change curve of volume fraction for the eutectic carbides.

Figure 4 illustrates the variation in volume percentage of eutectic carbides in MoVW2 and MoV steels for different holding times. The alloy element content in MoVW2 and MoV steels, separated from the C content, was 9.444% and 6.281%, respectively, as indicated in Table 1. Thus, it can be seen that the volume percentage of eutectic carbides in MoVW2 steel was higher than that in MoV steel. Additionally, the volume percentage of the eutectic carbides in both steels exhibited a decreasing trend with the increasing of holding time from approximately 0 to 80 h. The augmented migration rate of carbon and alloy elements was attributed to the prolonged holding time, which could promote the dissolution of the carbides. After holding for 80 h, the volume percentage of eutectic carbides in both steels was about 0.40%, which indicated that the content of eutectic carbides in both steels was relatively low at this holding time. In this regard, there was a slight difference in the variation in carbides between MoVW2 and MoV steels. In MoVW2 steel, the volume percentage of eutectic carbides was decreased from 3.31% to 1.50% after holding for 0~30 h, and then slightly increased by 0.10% after holding for 50 h. With the increase in holding time, the volume percentage of eutectic carbides was decreased to 0.38% after holding for 100 h. For MoV steel, the volume percentage of eutectic carbides also exhibited a decreasing trend, and it was decreased from 1.18% to 0.47% after holding for 0~100 h.

**Figure 5:**
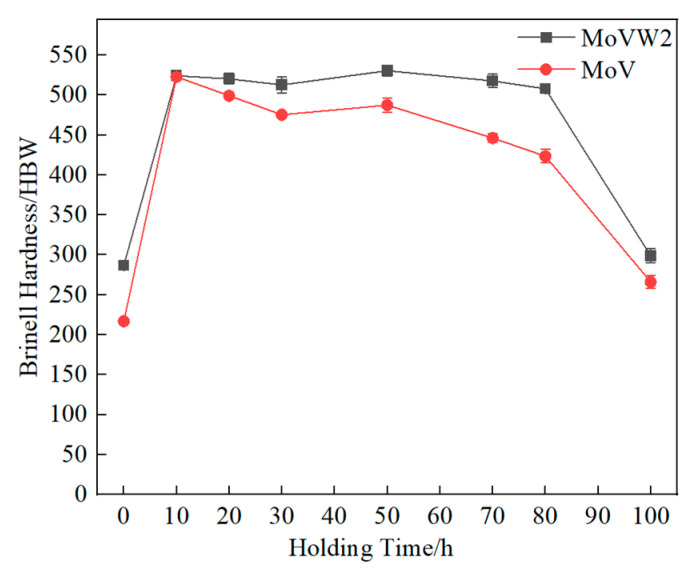
Variation in hardness for MoVW2 and MoV steels at different holding times.

Figure 5 shows the variation in Brinell hardness values with different holding times for MoVW2 and MoV steels. It can be seen that the hardness of MoVW2 steels increased from 286 to 524 HBW after holding for 0~10 h. During the heat treatment, the diffusion of carbides into MoVW2 steel matrix could cause the solid solution strengthening and the reduction of eutectic carbides, which was effective at increasing the hardness. After holding for 20~80 h, the hardness of the steels became stable. This was because the strengthening effect and the dissolution of carbides reached a dynamic balance. However, after holding for 100 h, the hardness rapidly decreased to 300 HBW, due to the grain coarsening and the decrease in the volume percentage of eutectic carbides. In addition, the hardness of MoV steel exhibited the same changing trend as MoVW2 steel. However, it had a higher hardness than that of MoVW2 steel, which was attributed to the higher content of W and Cr elements. The W element was effective at solid solution strengthening, and it could be combined with the C element to form different types of secondary carbides. It could hinder the movement of the dislocations and improve the performance of the steels [19]. Also, the Cr element contributed to the hardness of M_7_C_3_ carbides [20,21]. Conversely, the presence of bainite in MoV steel could reduce its hardness. Therefore, in the initial stage of holding, both steels exhibited a significant increase in hardness due to the dissolution of eutectic carbides and solid solution strengthening from secondary carbides. With the increase in holding time, the grain sizes were increased, resulting in less significant changes in the hardness. After holding for 50 h, the hardness was generally higher, which indicated that a long holding time was not unfavorable to the improvement of the hardness of the steels. In addition, the consumption of alloying elements by eutectic carbides could weaken the strengthening effect and reduce the precipitation-strengthening effect of eutectic carbides, resulting in a decrease in matrix strength and a noticeable decrease in hardness.

**Figure 6:**
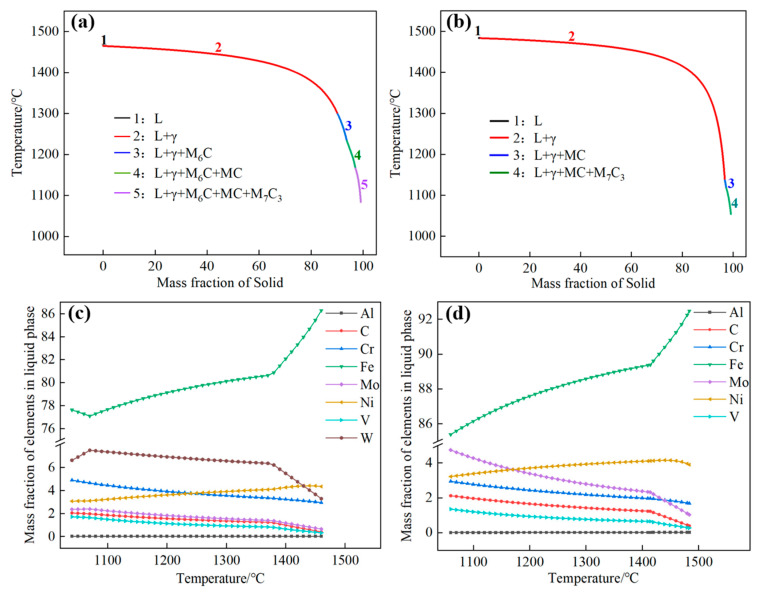
Solidification diagram of MoVW2 and MoV steels and elemental mass fraction in liquid phase as a function of temperature. (**a**,**c**) Nonequilibrium property diagrams of MoVW2, MoV; (**b**,**d**) Mass fraction of elements in liquid phase of MoVW2, MoV.

The researchers lacked a shared comprehension of the structure and reaction mechanism involved in the precipitation of eutectic carbides during the solidification of molten steel. However, it was generally believed that the eutectic carbides were formed near the endpoint during the solidification, and the formation of the composition of eutectic carbides had different phase constitution. Thus, Thermo-Calc software was utilized to investigate the non-equilibrium solidification phase diagrams in MoVW2 and MoV steels, as shown in Figure 6. The solidification sequence of MoVW2 steel is shown below:

L → L + γ-Fe → L + γ-Fe + M_6_C → L + γ-Fe + M_6_C + MC → L + γ-Fe + M_6_C + MC + M_7_C_3_

Apparently, during the solidification process, M_6_C, MC, and M_7_C_3_ eutectic carbides could be precipitated from the liquid phase at the solidification front. In addition, the solidification sequence of MoV steel is shown as follows:

L → L + γ-Fe → L + γ-Fe + MC → L + γ-Fe + MC + M_7_C_3_

During the solidification process, MC and M_7_C_3_ eutectic carbides could be precipitated from the liquid phase at the solidification front. Generally, MC eutectic carbides are carbides enriched in V, Ti, or Nb, and M_6_C eutectic carbides contain a large amount of Mo, Fe, and W, and small amounts of V and Cr elements. M_7_C_3_ eutectic carbides contain a large amount of Cr and Fe, and small amounts of V and Mo elements.

**Figure 7:**
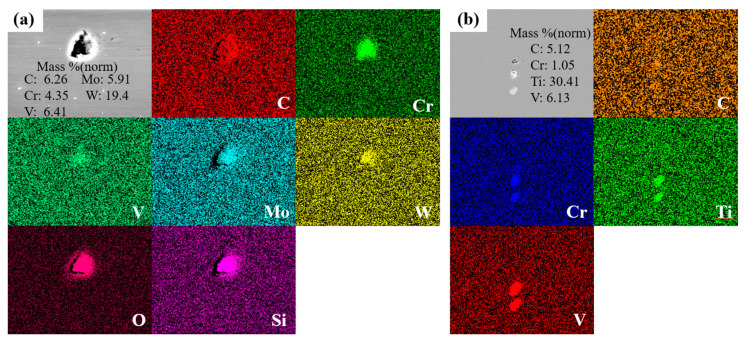
EDS element-mapping images for eutectic carbide: (**a**) MoVW2; (**b**) MoV.

Figure 7 shows the element-mapping analysis for the eutectic carbides. Through the EDX analysis of eutectic carbides after a long holding time, the atomic mass fraction of each element of the eutectic carbides of MoVW2 steel and MoV steel was obtained. It can be seen that the content of each element is different after the diffusion and dissolution of eutectic carbides. From Figure 7, it can be seen that the eutectic carbides in MoVW2 steel were enriched with Cr, V, Mo, and W elements, confirming that it was easy to form composite eutectic carbides such as MC, M_6_C, and M_7_C_3_. Additionally, the eutectic carbides in MoV steel were enriched with Cr, V, and Ti elements, belonging to composite eutectic carbides of MC and M_7_C_3_. During the early stage of solidification, the amount of C, Cr, Mo, V, and W elements in the liquid phase could be increased with the decreasing temperature due to the precipitation of M_6_C and MC eutectic carbides, along with the dissolution of a certain amount of Cr, Mo and other elements. The concentration of Mo elements exhibited a relatively stable condition during solidification; however, the concentration of Ni element was decreased due to the precipitation. The concentration of Al elements in the liquid phase were relatively low, and its variation trend was not significant.

The mass fractions of Fe and W elements in MoVW2 steel exhibited completely opposite trends. This could be because the W element was relatively stable during the early stage of solidification. As the solidification was carried out, the concentration of W element decreased due to the precipitation of M_6_C-type eutectic carbides containing W. During the prolonged solidification process of large-sized castings, the W element settled to the bottom of the liquid steel, resulting in its enrichment and providing compositional conditions for the formation of M_6_C carbides [22].

The high content of C and Cr in the liquid steel could promote the precipitation of Cr_7_C_3_ eutectic carbides. However, it could consume a large amount of alloying elements. Generally, the formation of MC carbides in steel was related to its purity. In the production process, there were small amounts of alloying elements such as V, Nb, and Ti, making the formation of (V, Nb, Ti) (C,N) eutectic carbides quite probable. Additionally, MC carbides often acted as heterogeneous nucleation sites, promoting the nucleation and refinement of M_7_C_3_ eutectic carbides’ size [23,24,25].

## 4. Conclusions

1. After high-temperature diffusion at 1230 °C for 0~100 h, the volume percentage of eutectic carbides in MoVW2 and MoV steels gradually decreased. Extending the holding time aids carbide dissolution, leading to a spherical shape and reducing the impact of large, irregular eutectic carbides on the steel. In MoVW2 steel, the volume percentage of eutectic carbides decreased from 3.31% to 0.38%, while in MoV steel, it decreased from 1.18% to 0.47%.
2. The hardness of both MoVW2 and MoV steels was relatively high after holding for 50 h, with MoVW2 steel at 524 HBW and MoV steel at 488 HBW. This was attributed to the solid solution strengthening caused by the dissolution of carbides and the decrease in eutectic carbides. However, after holding for 80~100 h at 1230 °C, the weakening of precipitation strengthening and grain coarsening could significantly result in a decrease in the hardness of both steels.
3. The high W content could promote the precipitation of M_6_C-type eutectic carbides in MoVW2 steel. Additionally, due to the enrichment of C and Cr in both MoVW2 and MoV steels, the preferential precipitation of M_7_C_3_ eutectic carbides occurred in the final stage of solidification from the liquid steel.

## Figures and Tables

**Table 1:** Chemical composition of the two Cr-Ni-Mo-V experimental steels (mass fraction, %).

Sample	C	Cr	Ni	Mo	V	W	Nb	Ti	Al	Fe
MoVW2	0.33	3.01	3.20	0.62	0.35	2.20	0.028	0.010	0.026	Bal.
MoV	0.36	1.82	3.12	1.10	0.22	/	/	0.003	0.018	Bal.

## Data Availability

Not applicable.
